# A Comparison of Prediction of Adverse Perinatal Outcomes between Hadlock and INTERGROWTH-21^st^ Standards at the Third Trimester

**DOI:** 10.1155/2019/7698038

**Published:** 2019-01-09

**Authors:** Chen Zhu, Yun-Yun Ren, Jiang-Nan Wu, Qiong-Jie Zhou

**Affiliations:** ^1^Department of Ultrasound, Obstetrics and Gynecology Hospital, Fudan University, Shanghai 200011, China; ^2^Department of Clinical Epidemiology, Obstetrics and Gynecology Hospital, Fudan University, Shanghai 200011, China; ^3^Department of Obstetrics, Obstetrics and Gynecology Hospital, Fudan University, Shanghai 200011, China

## Abstract

Little is known about the clinical value of the Hadlock and INTERGROWTH-21^st^ EFW standards for predicting adverse perinatal outcomes (APOs) in the third trimester. The purpose of this study was to study the association between low estimated fetal weight percentile (EFWc) in the third trimester and the risk of APOs and compare predictions of APOs between Hadlock and INTERGROWTH-21^st^ EFW standards. A prospective cohort of 690 singleton pregnancies with ultrasonography performed in the third trimester between March 2015 and March 2016 in China was conducted. EFW and the corresponding EFWc were measured using the Hadlock and INTERGROWTH-21^st^ standards, respectively. Cox proportional hazard models were used to assess the relationship between low EFWc (i.e., <5 percentile, P5) and the risk of APOs. Compared with fetuses with ≥P5 of the EFWc, fetuses with <P5 of the EFWc were much more likely to have an APO, with adjusted hazard ratios of 35.0 (95% confidence interval, 13.9-88.5) and 17.5 (7.7-39.6) for the Hadlock and INTERGROWTH standards, respectively. The Hadlock-EFWc had a higher predictive accuracy for APOs than the INTERGROWTH-EFWc, with area under the receiver operating characteristic curve of 0.94 (0.92-0.95) and 0.90 (0.87-0.92), respectively (P=0.007). The cutoff value for the INTERGROWTH-EFWc was percentile 11.61 with a sensitivity and specificity of 87.9% and 80.5%, respectively. For the Hadlock-EFWc, the corresponding sensitivity and specificity were 93.9% and 81.2%, with a cutoff value of percentile 8.65. Fetuses with low EFWc (i.e., <P5) were associated with an increased risk of APOs. APOs were more accurately predicted when EFWc was measured by the Hadlock standard than by the INTERGROWTH-21^st^ standard.

## 1. Introduction

Adverse perinatal outcomes (APOs) late in gestation are a major cause of fetal and neonatal deaths worldwide despite a substantial improvement in obstetric care over the past decades [1, 2]. However, the origins of the APOs vary and are mostly unknown, making the prediction difficult and limiting preventive action [3]. Using ultrasound to screen for fetuses with fetal growth restriction (FGR), a major determinant of APOs, is a strategy to identify pregnancies at a higher risk of APOs and is widespread in obstetric practice [4, 5]. Screening procedures for FGR need to identify small babies and then differentiate between those that are healthy and those that are pathologically small [4]. Assessment of fetal growth, such as an ultrasonographic estimated fetal weight (EFW), has been shown to be an effective method to reduce perinatal mortality in high-risk pregnancies [3, 6].

Hadlock et al. [7] introduced formulas to EFW with ultrasound measurements, and these have been widely used in China for decades. Recently, the INTERGROWTH-21^st^ Project, which was derived from an international, multicenter study of urban populations, established another EFW standard [8]. We previously compared the effectiveness of EFW values based on the Hadlock and INTERGROWTH standards for predicting the risk of FGR [9]. However, little is known about the clinical value of the Hadlock and INTERGROWTH-21^st^ EFW standards for predicting APOs in the third trimester.

Therefore, we conducted a prospective cohort study of singleton pregnant women to study the association between EFW percentile (EFWc) and the risk of APOs and compare the effectiveness of Hadlock-EFW and INTERGROWTH-EFW for predicting APOs. Based on the findings that a lower EFW is associated with the risk of FGR and reduction of perinatal mortality, we hypothesized that a lower EFWc measured in the third trimester may be associated with an increased risk of APOs and that EFWc-INTERGROWTH might have better predictive efficacy than does EFWc-Hadlock.

## 2. Materials and Methods

### 2.1. Study Population

In this prospective cohort study, singleton pregnant women attended their routine third-trimester antenatal examinations at 28, 32, 36, 38, and 40 weeks of gestation (within 1 week either side) at the Obstetrics and Gynecology Hospital of Fudan University, Shanghai, China, between March 2015 and March 2016. Pregnant women who had signed an informed consent document and completed the scans in the department of ultrasound of the hospital were sequentially enrolled in the study and followed up until delivery (the last case was completed in June 2016). Exclusion criteria included women who had refused consent and who had multiple pregnancies. Maternal characteristics, including maternal age (years) and parity (nulliparous or multiparous), were surveyed at the first measurement. Whether the pregnancies were conceived naturally or via assisted reproduction technique (ART) was self-reported by the pregnant women. Maternal complications, such as gestational diabetes (GDM) and gestational hypertensive disorders (GHD), were diagnosed based on the results of the oral glucose tolerance test (OGTT) and measurements of blood pressure and proteinuria. Pregnancy outcomes, including birth weight, fetal gender, and related APOs, were collected after delivery. The institutional ethics committee approved the study protocol (2017-24), and all patients provided written informed consent.

### 2.2. Measurements

A prenatal ultrasonographic examination with complete fetal growth measurements was performed for all participants. All ultrasound scans were conducted using an ALOKA Prosound *α*7 ultrasound device (Hitachi Medical, Tokyo, Japan) and a GE Voluson-E6 ultrasound device (GE Healthcare, Zipf, Austria). The scans were performed by two sonologists (C. Z. and Y.-Y. R.) with more than 10 years of experience in obstetric ultrasonography (more than 10,000 cases of fetal growth measurements). All ultrasound examinations followed the same protocols as those used in clinical practice [10]. Gestational age was in all pregnancies calculated on the basis of the measurement of fetal crown-rump length [11] at 11-13 weeks. In this study, all women underwent ultrasonography at 28, 32, 36, 38, 40 weeks of gestation (within 1 week either side). The following fetal growth measurements were obtained by ultrasonography: biparietal diameter (BPD), head circumference (HC), abdominal circumference (AC), and femur length (FL). Using the last fetal growth measurements before delivery, EFW and EFWc were calculated with the INTERGROWTH-21^st^ EFW standards [8] (henceforth referred to as INTERGROWTH-EFW and INTERGROWTH-EFWc, respectively): ln(EFW) = 5.084820 - 54.06633 × (AC/100)^3^ - 95.80076 × (AC/100)^3^  × ln(AC/100) + 3.136370 × (HC/100). Hadlock EFW and EFWc standards [7] were also measured on the basis of the last scan before delivery, as follows: log_10_EFW = 1.5662 - 0.0108 × (HC) + 0.0468 × (AC) + 0.171 × (FL) + 0.00034 × (HC)^2^ - 0.003685 × (AC × FL).

### 2.3. Definitions of Outcomes and Variables

APOs in the present study included a nonreassuring fetal status (NRFS) requiring emergency caesarean section, a 5-minute Apgar score of <7, neonatal metabolic acidosis, or stillbirth. NRFS was defined as an abnormal fetal heart rate tracing during antepartum and intrapartum monitoring [12]. Neonatal metabolic acidosis [13] was defined as UA pH <7.2 and base excess <-5 mmol/L in newborns. GDM was defined based on a fasting blood glucose level (BGL) ≥5.1 mmol/L, 1 h BGL≥10.0 mmol/L, or 2 h BGL≥8.5 mmol/L after a 75 g OGTT [14]. GHD was defined according to the Chinese Guidelines for the Management of Hypertensive Disorders in Pregnancy 2015 [15]. Gestational age at last ultrasound scan and at delivery was classified as two subgroups (< or ≥ 35 weeks for gestational age at last scan and < or ≥ 37 weeks for gestational age at delivery). Indications for caesarean section and for preterm delivery were in accordance with the guidelines of Chinese consensus guideline [16] and mainly included NRFS and/or severe preeclampsia (Table S1).

### 2.4. Statistical Analysis

Continuous data are expressed as the means ± standard deviation (SD), categorical data are expressed as n (%), and nonnormal variables were presented as the medians (25th and 75th) between groups of infants with and without APOs. Student's* t*-test was conducted to compare the means, and the Mann–Whitney* U* test was conducted to compare the medians, while the chi-square test or Fisher's exact test was used to assess proportions between the two groups. We derived categorical variables from the percentile of INTERGROWTH-EFWc and Hadlock-EFWc by the fifth percentile. Fetuses with a percentile of <5^th^ were grouped as high-risk, and those with a percentile of  ≥5^th^ were the control group. A Cox proportional hazards model was modeled to assess the relationship between fetal EFWc and the risk of APO and was presented as a hazard ratio (HR) and 95% confidence interval (95% CI). Potential confounders, such as maternal age (years), parity (nulliparous or multiparous), GDM (yes or no), GHD (yes or no), and ART (yes or no), were controlled in the adjusted models. Gestational age at the ultrasound scan (< or ≥ 35 weeks) was also included in the multivariable model to exclude potential bias of the differences between the APO and non-APO groups in terms of gestational age at ultrasound scan. However, gestational age at delivery (< or ≥ 37 weeks) was not included in the adjusted model because of a possible collinearity between gestational age at ultrasound and gestational age at delivery and the fact that the statistical model can't tolerate too many variables (the APO cases are limited in our study). Receiver operating characteristic (ROC) curve analyses were performed to evaluate the diagnostic value of the percentile of INTERGROWTH-EFWc and Hadlock-EFWc for predicting APOs. Cutoff values for APOs and the corresponding sensitivity and specificity were selected when the integrated area under the ROC curve (AUC) was statistically significant. The AUC of the percentile of INTERGROWTH-EFWc and Hadlock-EFWc on APO were compared using the Delong et al. [17] method. All other statistical tests were conducted using IBM SPSS Statistics version 22.0 (IBM Corp., Armonk, NY, USA).* P* values <0.05 were considered statistically significant.

## 3. Results

### 3.1. General Characteristics

**Figure 1:**
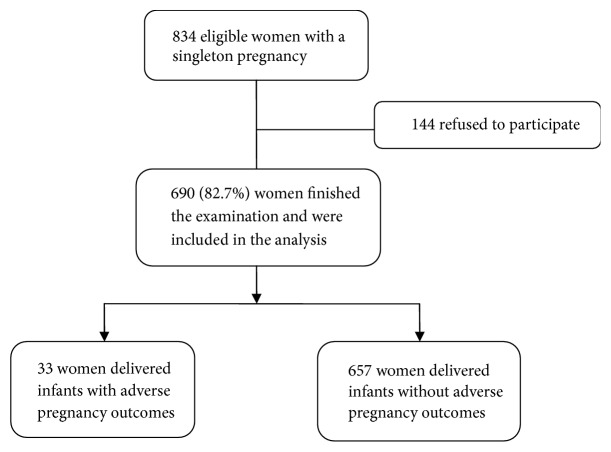
Flowchart of the study.

A total of 834 eligible women were identified. Among these women, 82.7% (690/834) provided written informed consent and were enrolled in the cohort (Figure 1). Among these subjects, 33 (4.8%) delivered infants with APOs, including NRFS requiring emergency cesarean delivery (n=29), 5-min Apgar <7 (n=9), neonatal metabolic acidosis (n=14), and NICU admission (n=8), perinatal death (*n*=0). All infants with APOs were delivered by cesarean section, including 22 cases because of NRFS, 7 cases because of maternal severe preeclampsia, and 4 cases due to NRFS and maternal severe preeclampsia (Table S1). The maternal and fetal characteristics, ultrasound markers in the third trimester, and perinatal outcomes observed in the groups of infants with and without APO are presented in Table 1. Pregnant women who delivered infants with APOs were more likely to be nulliparous, complicated with GDM and conceived by ART, than were women with births without APOs. Measurements of ultrasound markers were lower among pregnant women with infants with APOs than among women with infants without APOs. Compared with pregnant women who delivered infants without APOs, those who delivered infants with APOs had earlier gestational age for last ultrasound scan and delivery.

### 3.2. Association between a High Risk of Ultrasound Markers and the Risk of APO

Cox proportional hazards models showed that, compared with a fetus of ≥P5 on the INTERGROWTH-EFWc, infants with a high risk of <P5 were associated with an increased likelihood of APOs with unadjusted and adjusted HRs of 18.4 (95% CI: 8.9-38.0) and 17.5 (7.7-39.6), respectively. Similarly, a high risk of Hadlock-EFWc was related to the risk of APO with unadjusted and adjusted HRs of 35.0 (15.2-80.9) and 35.0 (13.9-88.5), respectively (Table 2).

### 3.3. Comparison between the Two Methods for Predicting APOs

**Figure 2:**
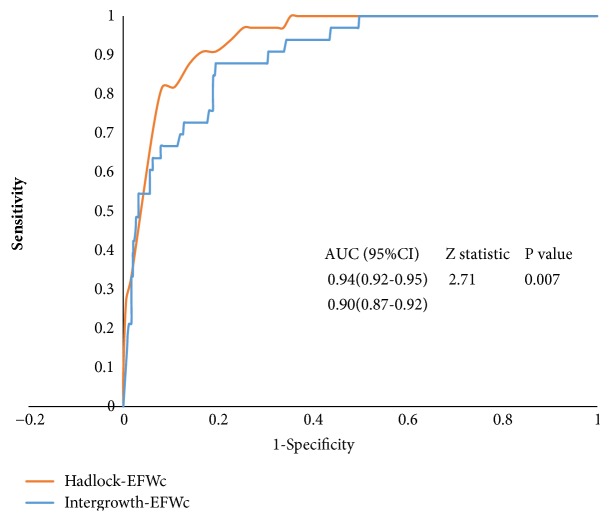
Receiver operating characteristic curve analyses comparing the predictive values of the EFWc in prevention of adverse perinatal outcomes between the Hadlock and INTERGROWTH standard method. AUC: area under the curve.

An ROC curve's analysis indicated that both percentiles of the INTERGROWTH-EFWc and Hadlock-EFWc had significant value for predicting APOs with an AUC of 0.90 (0.87-0.92) and 0.94 (0.92-0.95), respectively. The cutoff value of the INTERGROWTH-EFWc was 11.61 percentile (P11.61), with sensitivity and specificity of 87.9% and 80.5%, respectively. For the Hadlock-EFWc, the cutoff value was 8.65 percentile (P8.65), with sensitivity and specificity of 93.9% and 81.2%, respectively (Table 3). There was a significant difference in the AUC between the two methods (Z value=2.71,* P*=0.007) (Figure 2).

## 4. Discussion

In this prospective cohort study of 690 pregnant Chinese women, we found that EFW assessed in the third trimester by ultrasound scanning has high value for predicting APOs. Fetuses with a lower percentile of EFW (e.g., less than P5 of the EFW) were at higher risk of having an APO. In addition, when we compared the predictive value of EFWc for APOs between the Hadlock and INTERGROWTH standards, we found that, in Chinese fetuses, the Hadlock-EFWc was better at predicting APOs.

The findings presented here verify the necessity of ultrasound examinations in pregnant women in the third trimester because a fetus with a low EFWc measured in late pregnancy is at much higher risk of an APO. This association may be predominantly attributed to the relationship between a lower EFWc and the FGR, which are clear risk factors for APOs [3, 18, 19]. The cutoff values for INTERGROWTH-EFWc and Hadlock-EFWc (P11.61 and P8.65, respectively) were close to P10 in the ROC analyses, further supporting this notion because this is usually used as the definition of small-for-gestational age.

Unlike the Hadlock standard, which has been used in China to assess fetal size and monitor fetal growth since the 1980s, the INTERGROWTH-21^st^ standard was not released until recently [10]. The standard declares that “one size fits all” and is considered a new globally applicable standard because it was derived from an international, multicenter study of urban populations [20]. However, we found that the Hadlock-EFWc was superior to the INTERGROWTH-EFWc standard in predicting APOs. This finding suggests that the INTERGROWTH standard may be less compliant than the Hadlock standard in the Chinese population, a result that aligns with the findings of a previous study that found that using the INTERGROWTH standard led to a large number of fetuses being placed at risk of misdiagnosis with small fetal size [21]. When they compared results with a Canadian reference, Liu S et al. [22] found the positive skewness (left shift) of the EFWc distribution of the INTERGROWTH standard, which might reduce the sensitivity of the standard in screening small-for-gestational age and in predicting APOs. The difference between the two EFWc methods in the ability to predict APOs might be partly attributed to a parameter (FL) included in the Hadlock formula [7] that was thought to significantly improve estimates of fetal weight and to account for differences in fetal size among different races [23, 24].

The present study has several strengths. First, strict implementation of the inclusion and exclusion criteria, the high follow-up rate of the cohort, and good intra- and interoperator measurement repeatability minimize the possibility of selection and measurement bias. Second, we first explored and compared the predictive values of the EFWc measured by the Hadlock and INTERGROWTH standards for APOs, and our results provide evidence for the importance of using ultrasound examinations in the third trimester and the applicability of different criteria in Chinese fetuses. However, this study also has some limitations. The first limitation is that this was a single-center study, and the representativeness of the sample may therefore limit the generalizability of the results. Secondly, the overall cesarean section rate for the study cohort is 43.6% (301/690), which is higher than most delivery centers in western countries and might impact the generalization of the results, but it is basically in line with the reality situation of Shanghai (52.4%) in China [25]. Therefore, further studies that include multiple centers are needed to verify our findings.

In conclusion, in this single-center prospective cohort study of Chinese women, we found that a low EFWc measured in the third trimester was associated with an increased risk of APOs and that APOs were predicted better by EFWc measured by the Hadlock method than those measured by INTERGROWTH standard. Measuring the Hadlock-EFWc in the third trimester may therefore be useful for monitoring high-risk fetuses and providing better information for obstetric decision-making.

## Figures and Tables

**Table 1:** Parameter of ultrasound scan and maternal and pregnancy characteristics variables between the groups of infants with and without adverse perinatal outcomes.

Parameter/variable	Infants without APO (n=657)	Infants with APO (n=33)	*P *value

Ultrasound markers in the third trimester			
Gestational age < 35 weeks at last scan	47 (7.2%)	14 (42.4%)	<0.001^##^
INTERGROWTH-EFW (g)	2839.0 (2520.5-3088.6)	1930.0 (1210.4-2090.6)	<0.001^∗∗^
INTERGROWTH-EFW centile	32.1 (15.8-54.5)	1.9 (0.5-10.5)	<0.001^∗∗^
Hadlock-EFW (g)	2840.4 (2543.4-3099.1)	1926.8(1311.4-2137.8)	<0.001^∗∗^
Hadlock-EFW centile	29.0 (10.0-50.0)	3.0 (1.0-4.0)	<0.001^∗∗^
Maternal characteristics			
Maternal age (years)	30.1 (4.1)	30.7 (3.6)	0.34^∗^
Nulliparous	399 (60.7%)	19 (57.6%)	0.72^#^
Gestational diabetes mellitus	42 (6.4%)	4 (12.1%)	0.27^##^
Gestational hypertension	21 (3.2%)	13 (39.4%)	<0.001^##^
ART conception	10 (1.5%)	5 (15.2%)	<0.001^##^
Perinatal outcomes			
Birth weight (g)	3340 (3040-3590)	1990 (1490-2220)	<0.001^∗∗^
Male	302 (46.0%)	14 (42.4%)	0.14^∗∗^
Gestational age < 37 weeks at delivery	30 (5.0%)	16 (48.5%)	<0.001^##^
Cesarean delivery	268 (44.7%)	33 (100%)	<0.001^∗∗^

Continuous data were expressed as means ± standard deviation and categorical data as n (%).

APO: adverse perinatal outcome; ART: assisted reproduction technique.

^∗^ *P* value for Student's *t*-test; ^∗∗^*P* value for Mann–Whitney *U* test; ^#^*P* value for Chi-square test; ^##^*P* value for Fisher's exact test.

**Table 2:** Hazard ratios and 95% confidence interval for adverse perinatal outcomes among infants who had percentile of <5^th^ of the EFWc according to the INTERGROWTH and Hadlock method.

Groups of infants	Unadjusted model				Adjusted model^∗^			
	Hazard ratio	95% CI	Wald *χ*^2^ value	*P *value	Hazard ratio	95% CI	Wald *χ*^2^ value	*P *value
INTERGROWTH-EFWc								
≥ Percentile 5	1.0				1.0			
< Percentile 5	18.4	8.9-38.0	61.9	<0.001	17.5	7.7-39.6	47.1	<0.001
Hadlock-EFWc								
≥ Percentile 5	1.0				1.0			
< Percentile 5	35.0	15.2-80.9	69.3	<0.001	35.0	13.9-88.5	56.8	<0.001

^*∗*^Adjusted model controlling for maternal age (year), parity (nulliparous or multiparity), gestational diabetes mellitus (yes or no), gestational hypertension (yes or no), gestational age at last scan (< or *⩾* 35 weeks), and ART conception (yes or no).

**Table 3:** Comparison of predictive values for adverse perinatal outcomes between the INTERGROWTH and Hadlock EFWc.

Variable	Cutoff value (percentile)^∗^	Sensitivity (95% CI)	Specificity (95% CI)	Positive predictive value (95% CI)	Negative predictive value (95% CI)

INTERGROWTH-EFWc	11.61	87.9 (72.0-97.0)	80.5 (77.5-83.5)	18.5 (15.5-21.4)	99.2 (98.6-99.9)
Hadlock-EFWc	8.65	93.9 (80.0-99.0)	81.2 (78.3-84.3)	20.1 (17.1-23.2)	99.6 (99.2-100)

^*∗*^Fetus who has a percentile of EFWc of < the cutoff value was judged to develop at least one adverse perinatal outcome.

## Data Availability

The.xlsx data used to support the findings of this study are included within the supplementary information file(s).
