# Supplementary material for: A Comparison of Prediction of Adverse Perinatal Outcomes between Hadlock and INTERGROWTH-21st Standards at the Third Trimester
Source: Biomed Res Int. 2019 Jan 9;2019:7698038. doi: 10.1155/2019/7698038 (PMC6343179; doi:10.1155/2019/7698038)
Supplement: Supplementary Materials — The following supporting information may be found in the online version of this article. Table S1: characters of adverse perinatal outcomes (APOs). [file 7698038.f1.docx]

7698038: “A comparison of prediction of adverse perinatal outcomes between Hadlock and INTERGROWTH-21st standards at the third trimester”

**Supplementary Material Description**

**Table S1** The two standards estimated fetal weight percentile (EFWc) and clinical characters of 33 cases of adverse perinatal outcomes (APOs)

| Case No. | Hadlock  EFWc | INTERGROWTH  EFWc | Gestational week of delivery  (week) | Gestational week of the last scan  (week) | Birth weight (g) | EFWc | Type of APOs | Indication for emergency Cesarean delivery |
| --- | --- | --- | --- | --- | --- | --- | --- | --- |
| 1 | 2.7 | 0.5 | 28.5 | 28.5 | 780 | < P10 | emergency cesarean delivery; 5-min Apgar <7; NICU | Severe preeclampsia |
| 2 | <1 | <1 | 34.4 | 34.4 | 1250 | < P3 | emergency cesarean delivery; neonatal metabolic acidosis; 5-min Apgar <7; NICU | NRFS, severe preeclampsia |
| 3 | 17.8 | 27.6 | 38.3 | 37.3 | 2460 | =P3 | emergency cesarean delivery; | NRFS |
| 4 | 3.0 | 11.2 | 37.4 | 37.1 | 2200 | < P3 | emergency cesarean delivery; | NRFS |
| 5 | 1.0 | 0.5 | 36.2 | 35.4 | 1850 | < P3 | emergency cesarean delivery; | NRFS |
| 6 | 3.0 | 1.3 | 33.2 | 32.2 | 1620 | >P10 | emergency cesarean delivery | NRFS |
| 7 | 2.0 | 0.7 | 38.3 | 37.6 | 2240 | < P3 | emergency cesarean delivery | NRFS |
| 8 | 3.0 | 1.5 | 38.4 | 38.3 | 2170 | < P3 | emergency cesarean delivery | NRFS |
| 9 | 4.1 | 10.5 | 37.1 | 35.2 | 1990 | < P3 | neonatal metabolic acidosis; 5-min Apgar <7 | NRFS |
| 10 | 3.0 | 7.2 | 37.6 | 37.6 | 2400 | < P10 | emergency cesarean delivery; | NRFS |
| 11 | 1.0 | 0.3 | 33.2 | 32.1 | 1180 | < P3 | neonatal metabolic acidosis | Severe preeclampsia |
| 12 | <1 | <1 | 28.3 | 28.3 | 770 | < P10 | emergency cesarean delivery; neonatal metabolic acidosis; 5-min Apgar <7; NICU | Severe preeclampsia |
| 13 | 3.0 | 3.5 | 36.6 | 36.1 | 2080 | < P10 | emergency cesarean delivery; neonatal metabolic acidosis; 5-min Apgar <7; NICU | NRFS, severe preeclampsia |
| 14 | 3.0 | 1.1 | 29.2 | 28.5 | 950 | < P10 | emergency cesarean delivery; neonatal metabolic acidosis; 5-min Apgar <7; NICU | Severe preeclampsia |
| 15 | <1 | <1 | 37.6 | 36.5 | 1800 | < P3 | neonatal metabolic acidosis; 5-min Apgar <7; NICU | Severe preeclampsia |
| 16 | 1.0 | <1 | 33.3 | 33.1 | 1260 | < P3 | emergency cesarean delivery; neonatal metabolic acidosis; 5-min Apgar <7; NICU | NRFS |
| 17 | 3.8 | 4.1 | 37.3 | 36.6 | 2080 | < P3 | emergency cesarean delivery; neonatal metabolic acidosis; | NRFS |
| 18 | 1.0 | <1 | 37.2 | 37.2 | 1770 | < P3 | emergency cesarean delivery; neonatal metabolic acidosis | NRFS |
| 19 | 3.0 | 1.6 | 38.2 | 37.5 | 2090 | < P3 | emergency cesarean delivery | NRFS |
| 20 | 3.0 | 11.1 | 38.3 | 37.3 | 2360 | < P3 | emergency cesarean delivery | NRFS |
| 21 | 1.0 | <1 | 28.4 | 28.1 | 810 | < P10 | emergency cesarean delivery; 5-min Apgar <7; neonatal metabolic acidosis; | Severe preeclampsia |
| 22 | 3.0 | 1.1 | 29.2 | 28.5 | 950 | < P10 | emergency cesarean delivery; NICU | Severe preeclampsia |
| 23 | 3.6 | 11.5 | 37.3 | 37.3 | 2130 | < P3 | emergency cesarean delivery | NRFS |
| 24 | 1.0 | <1 | 38.3 | 37.3 | 2360 | < P3 | emergency cesarean delivery | NRFS |
| 25 | 5.7 | 4.9 | 37.3 | 35.5 | 2360 | < P10 | emergency cesarean delivery | NRFS |
| 26 | 3.6 | 3.6 | 37.1 | 36.4 | 2210 | < P3 | emergency cesarean delivery | NRFS, severe preeclampsia |
| 27 | 3.1 | 11.0 | 36 | 35.3 | 1920 | < P3 | emergency cesarean delivery | NRFS |
| 28 | 10.2 | 20.7 | 33.2 | 32.1 | 1490 | < P10 | emergency cesarean delivery | NRFS |
| 29 | 3.0 | 1.9 | 38.3 | 37.1 | 2590 | < P10 | emergency cesarean delivery | NRFS |
| 30 | 8.6 | 31.9 | 36.1 | 35.3 | 2220 | < P10 | emergency cesarean delivery; neonatal metabolic acidosis; | NRFS |
| 31 | 6.1 | 6.7 | 34.1 | 32.4 | 1690 | < P10 | emergency cesarean delivery; | NRFS |
| 32 | 3.0 | 1.9 | 38.3 | 38.3 | 2650 | < P10 | emergency cesarean delivery; neonatal metabolic acidosis; | NRFS |
| 33 | 7.1 | 18.8 | 34.3 | 34.3 | 1820 | >P10 | neonatal metabolic acidosis; | NRFS, severe preeclampsia |

NICU, neonatal intensive care unit; NRFS, non-reassuring fetal status.
